# Approaches to Asthma Diagnosis in Children and Adults

**DOI:** 10.3389/fped.2019.00148

**Published:** 2019-04-17

**Authors:** Sejal Saglani, Andrew N. Menzie-Gow

**Affiliations:** ^1^National Heart & Lung Institute, Imperial College London, London, United Kingdom; ^2^Department of Respiratory Paediatrics, Royal Brompton Hospital, London, United Kingdom; ^3^Department of Respiratory Medicine, Royal Brompton Hospital, London, United Kingdom

**Keywords:** asthma diagnosis, spirometry, exhaled nitric oxide, guidelines, inflammation, paediatric asthma, lung function, objective tests

## Abstract

Although the hallmark features of asthma include reversible airflow obstruction, airway eosinophilia, and symptoms of recurrent wheeze associated with breathlessness and cough, it is a heterogeneous disease. The extent of the pathophysiological abnormalities are variable between patients. Despite this, until recently, asthma diagnosis had been made very simplistically predominantly from a clinical history and examination, and often a trial of medication such as short acting bronchodilators. The limitations of this approach have become increasingly apparent with evidence of inappropriate over diagnosis, under diagnosis and misdiagnosis. Although there is no gold standard single test to make a diagnosis of asthma, there are several objective tests that can be used to support the diagnosis including physiological measures such as obstructive spirometry associated with bronchodilator reversibility and airway hyperresponsiveness. In addition, non-invasive tests of airway inflammation such as exhaled nitric oxide or peripheral blood eosinophils are important to identify those with an allergic or eosinophilic phenotype. Diagnostic guidelines reflect the importance of using objective tests to support a diagnosis of asthma, however practical application in the clinic may not be straightforward. The focus of this review is to discuss the need to undertake objective tests in all patients to support asthma diagnosis and not just rely on clinical features. The advantages, challenges and limitations of performing tests of lung function and airway inflammation in the clinic, the difficulties related to training and interpretation of results will be explored, and the utility and relevance of diagnostic tests will be compared in adults and children.

## Introduction

The essential components of a detailed history and examination remain central to making a diagnosis of asthma in both children and adults (1). Additional confirmative tests are recommended and will be discussed, but all guidelines emphasize the need to accurately establish the presence of a constellation of symptoms that align with asthma. This fundamental need to accurately identify a collection of symptoms that fit with asthma has recently been agreed by an expert consensus opinion from clinicians, researchers and scientists worldwide (2). It has been suggested that the term “asthma” should only be used as a descriptor that relates to a collection of symptoms. But no associated assumptions should be made about the underlying pathophysiological features driving the symptoms (2). It is suggested that this approach will prevent inappropriate treatments from being used and will encourage increased emphasis on individualized therapy. The focus of this review is to discuss the key features that constitute a diagnosis of asthma and to explore the role of objective confirmatory or supportive tests, highlighting the elements that are common to adult and childhood disease and some components that differ according to the age of the patient. The key components that contribute to a diagnosis of asthma include airway inflammation, hyperresponsiveness, bronchial obstruction, and symptoms. Each of these will be discussed, highlighting the relevance in children and adults and also the role of objective tests and the potential pitfalls that may lead to misdiagnosis.

## The Basics Need to be Correct Regardless of Patient Age

### History and Examination

Asthma is characterized by symptoms including wheeze, cough, breathlessness and chest tightness (3), all of which may fluctuate over time. The symptoms are common to children and adults, and an essential component is to obtain objective confirmation of symptoms either as documented doctor observed symptoms, or by administration of an objective questionnaire. A key issue that often leads to misdiagnosis in children is the mistaken assumption that all noisy breathing equates to wheeze and therefore asthma. Epidemiological data rely heavily on questionnaire reported symptoms, which may not always be accurate and may result in very varied reports of prevalence rates (4). However, for the individual patient, an accurate record of documented wheeze and symptoms consistent with asthma is critical to prevent inappropriate diagnosis, but equally importantly, inappropriate treatment (5, 6).

## Incorporating Objective Tests to Make a Diagnosis of Asthma: is it Necessary?

The importance of a correct diagnosis for the individual is obvious, however, equally important is the impact on cost to the health service of avoiding inappropriate prescription of asthma treatments. Application of a secondary screening programme, incorporating objective assessments of lung function and airway hyperresponsiveness, to a population who had a physician diagnosis of asthma, identified 28% of patients with a misdiagnosis (7) of whom 71% were on asthma medication. Moreover, the additional costs of the objective tests were significantly less than the costs of a lifetime of prescription of inappropriate medication. One-third of Canadian adults who had asthma diagnosed in the previous 5 years no longer had current asthma, likely because of an initial misdiagnosis (8). Factors contributing to the misdiagnosis of asthma include failure to confirm reversible airflow obstruction, the relatively poor sensitivity of spirometry alone to absolutely confirm asthma (especially in children), the day to day variability of symptoms and the numerous phenotypes of disease (9). Consequences of misdiagnosis not only include inappropriate treatment, but also lost opportunity and time in making a correct diagnosis to explain the patient's respiratory symptoms. It is important to remember that misdiagnosis incorporates both wrongly labeling another condition as asthma, but equally missing a diagnosis of asthma and failed treatment. Both have significant consequences (9). Given the availability of objective tests that can help to confirm the diagnosis and the potential unwanted effects of inappropriate or wrong diagnosis, many diagnostic algorithms now incorporate the need for objective tests in the diagnosis of asthma.

An important change in the approach to diagnosis has recently been introduced in England, where the National Institute of Health and Care Excellence (NICE), whose purpose is to generate evidence based and cost effective guidelines, has recently been published [https://www.nice.org.uk/guidance/ng80]. It was claimed by NICE that up to 1.2 million of the approximately 4 million people with asthma in the UK were misdiagnosed and therefore being prescribed wrong or inappropriate medication (10). For the first time in England, it has now been recommended that both spirometry and exhaled nitric oxide tests should be used in all patients older than 5 years to help in the confirmation of the diagnosis. This guideline has resulted in much debate and discussion especially because of differences from the British Thoracic Society and Scottish Intercollegiate National Guidance (BTS/SIGN) which have been used by clinicians in the UK for over 2 decades (11). In the context of asthma diagnosis, the big contrast between the two guidelines includes the implementation of objective tests as being absolutely central and essential for making a confirmed diagnosis in adults, and very important, and whenever possible, essential for a diagnosis in children over 5 years in the NICE guidance. Although the BTS/SIGN guidelines recommend the use of lung function tests to support asthma diagnosis, implementation of this to date has been very variable and limited.

## Tests to Assess Airway Inflammation in Asthma Diagnosis

### Use of Exhaled Nitric Oxide to Diagnose Asthma—In Adults

The NICE diagnostic algorithm for adults includes the need for an accurate history and physical examination, including wherever possible, objective confirmation of wheeze, however, the critical change at this point is the clear message that a diagnosis cannot be made only on symptoms, without an objective confirmatory test. The first objective test to be used in adults aged 17 years and over is exhaled nitric oxide. If a value of 40 parts per billion (ppb) or higher is measured in a patient with suspected asthma, this is considered a positive result, and is strongly supportive of asthma. However, situations in which exhaled nitric oxide may be low, despite the presence of asthma, are highlighted, the most important for adult patients being cigarette smoking (12).

The upregulation of nitric oxide (NO) by inflammatory cytokines in central and peripheral airways can be monitored in exhaled air. Increased fraction of exhaled NO (FeNO) reflects eosinophilic-mediated inflammatory pathways and likely steroid responsiveness moderately well in asthma (13). As the fundamental pathophysiology underlying asthma incorporates eosinophilic airway inflammation coupled with reversible airflow obstruction, exhaled nitric oxide is considered additive to measures of lung function as an indirect marker of airway inflammation. However, in addition to smoking, other factors, including atopy and current treatment with steroids influence measured exhaled nitric oxide. Therefore, although considered useful to help support a diagnosis of asthma, it is apparent that its clinical utility and accuracy is greatest for steroid naïve and non-smoking patients (14). It is because of the variability of exhaled nitric oxide that it must be remembered that although a high level is supportive of the diagnosis, a level below 40 ppb does not exclude asthma (15).

### Use of Exhaled Nitric Oxide to Diagnose Asthma—In Children

Although the NICE guidance includes objective tests for children to help confirm a diagnosis of asthma, in contrast to the adult diagnostic algorithm, exhaled nitric oxide measurement is not a required test for making the diagnosis in those under 17 years. Exhaled nitric oxide measurements are only recommended if there is diagnostic uncertainty after lung function tests and assessments of reversible airflow obstruction have been made [https://pathways.nice.org.uk/pathways/asthma#path=view%3A/pathways/asthma/assessing-and-diagnosing-asthma-in-under-17s.xml&content=view-node%3Anodes-diagnostic-uncertainty]. A systematic review of the utility of exhaled nitric oxide for the diagnosis of asthma in children has shown that the measure may be informative for a diagnosis when used in conjunction with other tests, but importantly, that the cut-off for normal should be lower in children than adults (16). Several factors, of relevance to children have been shown to influence levels of exhaled nitric oxide, including age, height, gender, race and passive smoke exposure (16). Another key issue for children, even if only considering those aged 5 and above, is their technique and ability to perform an adequate maneuver that allows maintenance of a sustained exhalation flow rate and an acceptable recording. With these numerous factors that affect values of exhaled nitric oxide in children, the American Thoracic Society (ATS) guidelines suggest values in children below 20 ppb are very unlikely to be associated with eosinophilic airway inflammation, whilst those above 50 ppb suggest airway eosinophilia and a response to corticosteroids (13). The ATS suggest values between 20 and 35 ppb should be interpreted in light of the clinical context, taking into consideration the various factors that may affect exhaled nitric oxide. Several pediatric studies have used 20 ppb as a cut-off and shown high sensitivity (86%), specificity (89%), positive (92%), and negative (80%) predictive value for asthma in children (17, 18). A significant factor that must be considered for children when interpreting values of exhaled nitric oxide is the influence of atopy. Allergic sensitization alone, without any clinical manifestation of atopic disease or asthma is strongly associated with elevated levels of exhaled nitric oxide (19, 20). Moreover, there is an association between elevated exhaled nitric oxide and current exposure to the allergen that the child is sensitized to and multiple sensitization may result in higher exhaled nitric oxide levels (20, 21). Despite the data from the ATS guidelines suggesting a value >20 ppb may be of clinical relevance in children, the abnormal cut-off level that has been set in the NICE guidelines for children is above 35 ppb for those aged 5–16 years. The test is only recommended in those children with diagnostic uncertainty after initial assessment and those with normal spirometry, or airflow obstruction without evidence of reversibility. On balance, an assessment of exhaled nitric oxide is helpful in supporting a diagnosis of asthma in children aged 5 and above, providing the challenges associated with technical ability to perform the test and the factors that may either elevate or lower the value are considered (Table 1). As a result, exhaled nitric oxide is currently predominantly used in specialist centers, where the equipment is used frequently, technical expertise in obtaining measurements is reliable, where children with diagnostic uncertainty are seen, and results are interpreted in the context of the influencing factors.

**Table 1:** Factors, independent of asthma, influencing exhaled nitric oxide levels.

	**Elevated/lower level**
Age	Increases with age, lower normal values in children than adults
Height	Increase in taller children
Ethnicity	Higher in Black than Caucasian children
Smoke exposure (passive/active)	Lower with smoke exposure
Allergic sensitization	Higher in atopic patients
Gender	Higher in males
Respiratory infection	Lower with concurrent infection
Technique—maintaining exhalation flow rate	Inaccurate results if flow not maintained
Consumption of nitrite containing foods, caffeine, alcohol	Increased values

### Sputum Eosinophils

Currently, assessment of airway eosinophils is not a requirement for the diagnosis of mild to moderate asthma. The utility of induced sputum inflammation is predominantly recommended for patients thought to have severe disease (22). In practice, the use of sputum eosinophils to make an asthma diagnosis is challenging because of the time and expertise required for both the induction, processing and analysis of the sample. For this reason, the utility of less invasive biomarkers that may reflect airway eosinophilia are preferred. A meta-analysis of the diagnostic accuracy of minimally invasive biomarkers (exhaled nitric oxide, blood eosinophils, total serum IgE) has shown each of these markers only moderately reflect sputum eosinophils with a sensitivity and specificity of 0.66 and 0.76 for exhaled nitric oxide, 0.71 and 0.77 for blood eosinophils (23). These data highlight that no single biomarker accurately reflects airway eosinophilia and if used alone, there is a substantial risk of both false positive and false negative diagnoses. Overall, it appears that blood eosinophils and exhaled nitric oxide have similar accuracy in reflecting sputum eosinophilia, regardless of the asthma phenotype, while serum IgE is less accurate (24). Given the difficulty in obtaining sputum samples and the restriction of its use to specialist respiratory centers, currently an assessment of sputum eosinophils is not routinely undertaken to make a diagnosis of asthma in adults or children.

In adult patients the main use of sputum eosinophils has been to guide management and tailoring of therapy to achieve a reduction in exacerbations, rather than to make the diagnosis of asthma (25). Unfortunately, to date, these results have not been reproduced in children, perhaps because of the longitudinal variability of sputum eosinophils counts within patients over time (26).

### Blood Eosinophils

A blood test is easier and can routinely be performed in all clinical settings, thus making the utility of peripheral, rather than airway eosinophils, more attractive to help make a diagnosis of asthma for both adults and children.

In children, several factors need to be considered prior to the interpretation of a blood eosinophil count. Firstly, the cut-off for normal values change with age. The range for blood eosinophils in healthy children aged between 6 months and 13 years is between 500 and 700 cells/mcl (27). Thus, using cut-offs of >300/mcl as suggested in numerous adult studies may be entirely inappropriate. Another factor that must be considered in children is the presence of atopic disease without asthma which may result in elevated blood eosinophils without airway eosinophilia. Therefore, if blood eosinophils are relied upon as a biomarker in a child with eczema and wheeze, disentangling the reason for peripheral eosinophilia is difficult and may lead to a false positive diagnosis (28). Another issue is the impact of steroid treatment on peripheral eosinophil count. If a child is steroid naïve, an elevated blood eosinophil count may truly represent airway eosinophilia, but for children on inhaled corticosteroids, the peripheral eosinophil count may be low or normal, while an airway eosinophilia may persist, this is especially true for children with severe asthma (29). On balance, in school-age children with asthma, given the number of potential caveats that may give a result that does not truly reflect airway eosinophils and without a cut-off for the upper limit of normal yet being established, there is currently no evidence to support the use of blood eosinophils as a diagnostic marker for asthma. The utility of blood eosinophils in preschool children with wheezing to predict asthma development and response to inhaled corticosteroids, has been better evaluated and will be discussed below in the section on preschool wheeze and diagnostic markers. Another caveat to the use of peripheral blood eosinophils in children, is the data showing correlations with airway eosinophils are based on values during stable disease, not during exacerbation. It is unclear whether peripheral blood eosinophils would be helpful in making a diagnosis of asthma during an exacerbation, especially in children, since so many acute attacks are driven by infection, when peripheral or airway inflammation may be predominantly neutrophilic, not eosinophilic.

Unlike the paucity of data supporting the utility of blood eosinophils for a diagnosis of asthma in children, there is significant evidence in adults that blood eosinophils are useful to identify those patients who are more likely to respond to specific therapies such as steroids or the anti-eosinophilic monoclonocal antibodies, Mepolizumab (30) and Benralizumab (31). However, it must be remembered that elevated blood eosinophils only reflect a particular phenotype of asthma, that which is predominantly driven by Th2 mediators and is likely to be steroid responsive. Therefore, an absence of peripheral eosinophilia does not exclude asthma. The need to consider symptom pattern and lung function to help support a diagnosis remains important. The use of blood eosinophils in primary care and even as a point of care test is becoming increasingly feasible, whereby a finger prick point of care device has been shown to have close correlation with differential cell counts obtained from samples by venepuncture (32). However, the most important clinical message when interpreting blood eosinophils for asthma diagnosis, is very high counts (>500 cells/mcl) have a high certainty of an associated airway eosinophilia, but for values <410 cells/mcl the relationship between blood and airway eosinophils becomes less clear and it is important to consider the overall clinical picture and all possible factors that might affect blood eosinophil counts (30).

## Lung Function Tests and Asthma Diagnosis

### Use of Spirometry to Diagnose Asthma—In Adults

Perhaps the most easily accessible test that can be used to support a diagnosis of asthma is spirometry. The presence of an obstructive picture, with a ratio of FEV_1_/FVC <70%, and associated reversibility following administration of bronchodilator is in keeping with asthma. Although it is assumed by most that spirometry is used to help confirm a diagnosis of asthma in adults, its use in primary and secondary care settings is limited. For this reason, the NICE guidelines that have been recently published in England include spirometry as a “must do” objective test for all patients over 5 years old with a suspected diagnosis of asthma (https://www.nice.org.uk/guidance/ng80). If an obstructive spirometry result is present, then it has been recommended that all adults, aged 17 and over, should undergo a bronchodilator reversibility test, and an improvement of ≥12% in FEV_1_ and an increase in ≥200 ml is considered a positive test of reversible airflow obstruction. However, one of the key issues about the use of spirometry to help diagnose asthma is the absolute requirement for its correct use and interpretation (15). If this is not done, there is a significant risk of both under and over diagnosis (33). Interpretation of spirometry results varies even between specialist lung function laboratories, with lack of standardization in relation to definitions for the lower limit of normal (34). Another issue that affects the interpretation of spirometry in the context of an asthma diagnosis is that values may be normal when assessed during stable disease in the clinic. The majority of adults seen in primary care have mild disease with well-preserved lung function. Airflow obstruction defined as a ratio of FEV_1_/FVC <70% was found in only 21% of adult patients diagnosed with asthma in a primary care setting (35). In the case of normal spirometry results, the NICE guidelines suggest a two to 4 week period of peak flow monitoring and more than 20% variability in results as a positive test supportive of asthma. The American Thoracic Society and the National Asthma Education and Prevention Program, both recommend the use of spirometry for the diagnosis of asthma, these recommendations have been in place since 2007. However, an assessment of the implementation of this recommendation by physicians for patients with newly diagnosed asthma demonstrated that only 47% had spirometry performed within a year of the diagnosis being made (36). Moreover, rates of use of spirometry in primary care were only 23% and 78% of patients were prescribed asthma drugs without previous spirometry (36). Therefore, despite recommendations and guidelines having been introduced for over a decade, implementation and physician practice seems to have remained largely unaffected. This pattern seems consistent across countries, with approximately 50% of patients with a diagnosis of asthma ever having had spirometry in a secondary care or specialist setting, and this value reducing to approximately 25% in primary care (37, 38). Overall, there is agreement among respiratory physicians that spirometry is important in making a diagnosis of asthma in adults. However, in practice, it is difficult to find evidence that the guidelines are being consistently implemented. A combination of physician education to undertake spirometry to the required standards, emphasis of its importance and providing knowledge in interpretation and importantly, resources and incentives to undertake the test from primary through to specialist care, is likely the only way it will be adopted more widely (Table 2).

**Table 2:** Advantages and challenges of the use of spirometry in making a diagnosis of asthma in adults and children.

**Advantages**	**Challenges**
Objective evidence of obstructive airways disease	Not reliable if technically inadequate maneuvers performed—skills to undertake procedure and calibrate and maintain equipment needed
Demonstration of variable airflow obstruction if bronchodilator reversibility also applied	Defined and agreed values for lower normal limit needed
Minimizes over diagnosis and misdiagnosis	Normal values do not rule out asthma—tests of airway hyperresponsiveness may be needed
Demonstration of reversible airflow obstruction provides objective evidence of asthma	Requires cooperation, cannot be reliably performed in children under 6 years, except in specialist centers
	Difficulties around agreement of lower normal limit—need to use reference values that allow for ethnicity
	Technical expertise in obtaining a satisfactory result is needed. Often best results obtained with incentive devices, which may not be available in primary care

### Use of Spirometry to Diagnose Asthma—In Children

Although confirmation of reversible airflow obstruction is as important for a diagnosis of asthma in children as in adults, the practical application of spirometry in children is even more of a challenge (Table 2). This applies to all levels of care from primary to specialist, because of the significant challenges of technical expertise required to undertake reliable and reproducible tests in children and the training and education needed by physicians in the interpretation of the data. These issues are highlighted by American data, which shows that although 52% of physicians who provided primary care to children used spirometry, only 21% used spirometry according to the national guidelines and only 35% of physicians surveyed were comfortable interpreting the test results (39). In addition, 21% of spirometry readings were interpreted incorrectly (40), emphasizing the need for training and quality control prior to use of spirometry for children in primary care. The challenges of using spirometry to diagnose asthma in children and the improvements that can be made following training and education of healthcare staff have been discussed in detail elsewhere (41). Another critical issue for children, even more prevalent than in adults, is that when measured during stable disease, spirometry is frequently normal, even in those with severe disease (42). The significant issues that arise when relying on spirometry in children to make a diagnosis of asthma have been highlighted recently in a study that assessed the usefulness of the recent NICE guidelines in England to accurately make a diagnosis of asthma when applied to a cohort of children being regularly followed. Only two of 89 children aged between 13 and 16 years, who were symptomatic met the definition of asthma when assessed according to the NICE diagnostic algorithm (FEV_1_:FVC <70%), but neither met the epidemiological, questionnaire based definition of asthma (43). Although a total of 10 children had FEV_1_:FVC <70%, 8 of those did not have symptoms consistent with current asthma (43). The risks of using cut-offs for airway obstruction and lower normal limits extrapolated from adult studies have been highlighted as a significant pitfall for the application of the pediatric NICE guidelines in clinical practice (44).

As asthma is characterized by variable airflow obstruction, a reduced FEV_1_/FVC ratio may not be present at all times, therefore, if clinical suspicion remains, spirometry may need to be repeated to demonstrate obstruction. Another way of recording variation in airflow obstruction is to undertake several measurements of peak expiratory flow rate (PEFR) at home for 1–2 weeks. Variation of >13% suggests variable airflow obstruction. However, correct technique must be ensured, and this relies on patient cooperation and adherence in undertaking the measurements (45). Demonstration of improvement in spirometry following bronchodilator may be more sensitive in children than detection of obstruction (46). Demonstration of reversibility after bronchodilator had good specificity for an asthma diagnosis (73%), but using a cut-off of 12% improvement for children carried poor diagnostic sensitivity (35%), while a cut-off of 8% was significantly better (47). Therefore, although demonstration of bronchodilator reversibility is important to help confirm a diagnosis of asthma, but it may not be appropriate to use a strict cut-off for improvement in children.

### Reference Values for Spirometry: the Global Lung Function Initiative (GLI)

Global, multi-ethnic all age reference equations are now available and have been endorsed by all major international respiratory societies (48). These are now considered the international gold standard and offer a unified approach to the interpretation and presentation of FEV_1_ and other spirometry measures. It is essential that these reference equations are used, but specifically in the context of demonstrating airway obstruction in children, it must be remembered that older reference equations may produce lower predicted values, which result in an over-estimation of lung function when interpreted as %predicted. Therefore, the switch to GLI-2012 will result in lower median FEV_1_ %predicted values overall, as well as age-specific and individual patient differences. Data for airway obstruction should be interpreted using the GLI-reference values as recommended by the NICE guidelines.

## Persistent Airflow Limitation and Asthma Diagnosis

An important sub-group of patients thought to have asthma may have obstructive spirometry, but without evidence of bronchodilator reversibility. These patients have a post-bronchodilator FEV1 and/or an FEV1/FVC less than the lower limits of normal, or persistent airflow limitation (49). Although this is unusual in asthma, it may still be consistent with the diagnosis. A multi-center study of children with persistent airflow limitation on spirometry, showed 93% of all children had a diagnosis of asthma, this was even after other diagnoses and co-morbidities had been excluded (49). Similarly approximately one-third of adults screened in asthma clinics have been shown to have persistent airflow obstruction (50). Those with persistent airflow limitation may have more severe disease, but the most important element prior to labeling these patients as asthma is to refer to a specialist center to ensure other diagnoses have been excluded.

In adults, the most common airway disease associated with fixed airway obstruction is chronic obstructive pulmonary disease (COPD). However, it has now been proposed that there is a gray area of overlap between patients that have asthma (with reversible airflow obstruction) and those with COPD and fixed airflow obstruction. This has led to the diagnostic label asthma-COPD overlap (ACO). Although the term has been proposed as a valid entity, the definition and clinical features of patients that may fit this category have remained uncertain. It appears to be characterized by persistent airflow limitation with several features usually associated with asthma and several features usually associated with COPD. There has been a published consensus definition which includes persistent airflow limitation in symptomatic individuals 40 years of age and older, a well-documented history of asthma in childhood or early adulthood and a significant exposure history to cigarette or biomass smoke (51). Overall, the diagnosis of ACO in adults remains uncertain and a challenge (52). This entity adds to the confusion, however, it brings back the importance of identifying a constellation of symptoms and looking for evidence of underlying pathophysiology, with the approach of targeting therapy in individuals according to “treatable traits” (2). Certainly, in children, the approach taken is that the presence of persistent airflow limitation in a child with presumed asthma should lead to referral to a specialist center and the search for alternative diagnoses. However, if no other diagnoses explain the child's symptoms and presentation, asthma with persistent airflow limitation is the diagnosis and the patient should be treated as such. It is important to remember, however, that therapy escalation to try to reach “normal” lung function may not be appropriate, especially if a child does not have evidence of eosinophilic airway inflammation. A trial of steroids to try to establish optimal lung function may be appropriate, but if there is no improvement and the child's symptoms are controlled, attempts to escalate steroid medication must be resisted in this exceptional scenario (53).

## Utility of Lung Function Tests Other Than Spirometry for Asthma Diagnosis

The limitation of spirometry in children is prevalent because of the reliance on the ability of the child to adequately perform voluntary maneuvers. This is a particular challenge in the absence of computer aided incentive devices (54). Alternative effort independent lung function tests can therefore be used in children, but none of these are available in primary or secondary care and issues around technical competence and normal values are even more of an issue than for spirometry, even in specialist centers. As a result, the application of tidal breathing or effort independent tests is currently limited predominantly to the research setting and being tested mainly in preschool children being assessed at specialist centers. The assessment of airway obstruction in children with severe asthma has been reviewed elsewhere (55) and the utility of forced oscillation technique and impulse oscillometry for asthma diagnosis have also been reviewed (56).

Other lung function tests that can be used include plethysmography, multiple breath washout (to measure lung clearance index) and airway resistance using the interruptor technique (Rint). However, none of these are used routinely in clinical practice, certainly none are available for use in primary or secondary care. The test used most commonly in specialist centers is plethysmography, however this measures lung volumes, and the role of assessing lung volumes in asthma diagnosis remains controversial. Although there is evidence that lung volumes may be complementary to spirometry to assess asthma severity (57), plethysmography alone is not routinely used to make the diagnosis. Similarly, the other tests have been used in small studies, especially in populations where voluntary maneuvers cannot be reliably performed, but currently their use remains limited to research and specialist centers, without obvious evidence for their role in routine clinical practice, in either children or adults.

## Assessment of Airway Hyperresponsiveness to Diagnose Asthma

As spirometry is often normal during stable disease in patients with a clinical picture consistent with asthma, an indirect bronchial provocation test to assess airway hyperresponsiveness can be used to help diagnostic confirmation. Various challenge agents can be used to induce hyperresponsiveness including histamine, methacholine, allergens, adenosine, and mannitol. However, the importance of optimal delivery of the inhaled agents to the airways to ensure reproducible data are generated has been highlighted (58). Inhaled methacholine, a direct cholinergic agonist, to evoke concentration-dependent airway smooth muscle contraction can be used and bronchoconstriction at low concentrations of methacholine (typically <4 mg/mL) suggest increased airway hyperresponsiveness. A novel method to report responsiveness using dose rather than concentration has recently been proposed (59). However, allergen challenge tests can only be undertaken in specialist centers and so are not routinely used for asthma diagnosis, but should be undertaken in patients with a symptom constellation suggestive of asthma, without evidence of reversible airflow obstruction or eosinophilic airway inflammation and without response to therapy.

In addition to inhaled challenge tests, another scenario that may uncover bronchoconstriction, and may also be supportive of an asthma diagnosis, is exercise. Exercise is a frequent precipitant of asthma symptoms, however an exercise test is of particular importance if this is the only trigger for symptoms in order to obtain objective confirmation of true bronchoconstriction and diagnose asthma, (60) and to exclude any contributory upper airway symptoms including exercise induced laryngeal obstruction which may be prevalent in both children and adults (61, 62). Importantly, the presence of exercise induced bronchoconstriction alone may not equate to a diagnosis of asthma as it may occur in people without asthma, the need to assess the complete clinical picture and airway inflammation remains essential.

## Assessments of Atopy in Asthma Diagnosis

Often a diagnosis of asthma is made clearer in the presence of associated allergic diseases including eczema, allergic rhinitis or food allergy. In children, allergic asthma is the most common phenotype (63) and therefore assessments of allergic sensitization, in the absence of disease manifestation, are undertaken to help support the diagnosis. However, what remains uncertain is how best to include tests of allergic sensitization into diagnostic algorithms for asthma (64). The presence of allergic sensitization, particularly to aeroallergens is supportive of the diagnosis, but the absence of sensitization does not rule out the disease. In school children, non-atopic asthma is rare and should be diagnosed after careful exclusion of other differential diagnoses (65).

The phenotype of “adult-onset asthma” is often non-atopic (66) and therefore atopy is not as central to the diagnosis in adults as in children. However, it must be remembered that atopy is not an all or nothing phenomenon and must itself be quantified in terms of severity (67). An absence of elevated specific IgE to a limited range of allergens may not exclude atopy, moreover, low peripheral IgE levels may not reflect low pulmonary mucosal IgE levels as has been shown by a reduction in bronchial IgE and associated improved lung function following omalizumab therapy in “non-atopic” adult asthmatics (68). As for blood eosinophils and other objective tests, the presence of allergic sensitization and indeed associated clinical manifestation of allergic diseases certainly supports a diagnosis of asthma in a patient with the correct symptom constellation, but the absence of atopy does not exclude asthma.

## Should we Have Diagnostic Algorithms/Guidelines for Asthma?

Unfortunately, we do not have a gold standard confirmatory test for asthma, so although objective markers may be used, they still all only provide evidence that is supportive of, or less indicative of asthma. Ultimately, a diagnosis at present can only be made based using a constellation of clinical features, supportive objective tests and frequently an assessment of response to therapy. The absence of a gold standard test, and the recognition that asthma constitutes a disease with an array of etiologies, phenotypes and clinical manifestations has led to the proposal that we should now diagnose “asthma” based on symptoms, and then identify pathological and physiological “treatable traits” to allow targeted and personalized therapy. Although in England the NICE guidelines have attempted to overcome the lack of utility of any objective tests to make a diagnosis of asthma, they remain restrictive, as the objective tests used assume the presence of predominantly eosinophilic airway inflammation and reversible airflow obstruction are required for the diagnosis. This approach has the risk of being too restrictive and potential for under diagnosis as numerous phenotypes and endotypes of the disease display either non-eosinophilic inflammation, or airflow limitation without obstruction. The inclusion of objective tests is important, and it should be remembered that a single test may not be enough to make a diagnosis, and a combination of tests demonstrating variable airflow obstruction and airway inflammation are needed. When applied to a cohort of 13–16 year old children spirometry, bronchodilator reversibility and exhaled nitric oxide were all normal in 24 of 56 children (43%) with current asthma defined epidemiologically (physician diagnosed asthma, current wheeze and prescribed asthma medication) (43). Moreover, the data question the cut-offs proposed for obstructive spirometry in children, the order in which the lung function tests are proposed and the position of bronchodilator reversibility within the algorithm. The authors plainly state the NICE algorithm should not be used for an asthma diagnosis in children until better evidence of its utility is available (43).

## Differential Diagnosis

Even though patients may present clinically with acute symptoms of breathlessness and wheeze, the long list of differential diagnoses that may cause these symptoms must be considered before asthma is diagnosed (69). Features in the history that are supportive of asthma include a positive family history in a parent or sibling, a history of atopy, including eczema, allergic rhinitis or food allergy and, for children, a symptom pattern that incorporates symptoms with several triggers such as exercise, cold air as well as upper respiratory infections. The important “red flags” in the clinical history and examination that should question the diagnosis in both children and adults have been clearly summarized in the British Thoracic Society/ Scottish Intercollegiate Network Guidelines (https://www.brit-thoracic.org.uk/document-library/clinical-information/asthma/btssign-asthma-guideline-quick-reference-guide-2016/). The initial structured clinical assessment should be used to help decide whether asthma is of high, intermediate or low probability. If any doubts about the diagnosis arise, it is essential that referral to a specialist is made prior to any diagnostic label being applied as the high risk of misdiagnosis and over diagnosis with the resulting undesirable consequences to the patient and inappropriate use of resources has become increasingly apparent (8).

## Should a Trial of Treatment be Used to Diagnose Asthma?

Given the difficulties associated with the absence of a gold standard diagnostic test for asthma, and especially in children, difficulties around appropriate lung function tests and assessments of airway inflammation, it has been argued that the only way to confirm the diagnosis is to assess response to a trial of asthma medication. However, this approach carries a significant risk of a misdiagnosis with the associated problems of inappropriate therapy. This is even more of a concern now since the BTS/SIGN guidance for the management of asthma suggests as required short acting bronchodilators should not be the starting point of treatment, but all patients should be commenced on anti-inflammatory treatment with low dose regular inhaled corticosteroids (70). Whenever possible, the diagnostic tests that have been outlined thus far to try to identify “treatable traits” such as evidence of eosinophilic airway inflammation and reversible airflow obstruction should be undertaken. This applies equally to children and adults. The concern is that a response of symptoms to a trial of therapy does not make a diagnosis. Indeed, a trial of therapy is not part of the diagnostic algorithm proposed by NICE in England (71). If a child or adult presents acutely with wheeze, then it is a priority to treat the symptoms and not wait for objective tests. However, once symptoms have been controlled, even if empirical maintenance therapy such as inhaled corticosteroids have been commenced, it remains important to undertake some objective testing once the patient is stable to help to confirm the diagnosis.

## Specific Considerations

### Diagnosing Asthma at the Extremes of Age Preschool Wheeze

Making a diagnosis of asthma in preschool children is a huge challenge because objective tests of airway inflammation and lung function are either invasive (broncho-alveolar lavage for inflammation) or require voluntary maneuvers and cooperation (spirometry). In addition, children under 5 years old have phenotypes of wheezing that are distinct from allergic asthma. Some only wheeze with respiratory infections in acute episodes and others may have persistent symptoms during and in between episodes. The diagnostic term preschool wheeze is therefore used in preference to asthma for children under 5 years old. Guidelines and recommendations for diagnosis and management have been published (72, 73). Diagnosis currently relies predominantly on confirmation of wheeze (doctor diagnosed wheeze), history and symptom pattern (which relies on accurate parental recall) and tests of atopy. However, the pitfalls of relying predominantly on subjective markers to make a diagnosis and direct management have been highlighted (74). Increasing efforts are being made to identify biomarkers to help guide management in preschool wheezers. The most promising is recent evidence of children with aero-allergen sensitization and elevated blood eosinophils as differential responders to maintenance inhaled corticosteroids (75). The role of respiratory infection (both viral and bacterial) and neutrophilic airway inflammation in mediating symptoms is being increasingly recognized in this age group (76), but biomarkers that distinguish children with predominant eosinophilic and allergic airways disease compared to those with infection driven disease are currently lacking.

### Asthma in the Elderly

It is important to recognize the impact of aging on lung physiology when diagnosing asthma in the elderly. Specifically, because aging impacts respiratory mechanics, the fixed threshold of <0.70 for the ratio FEV_1_ to FVC frequently misclassifies normal-for-age spirometry as airflow obstruction. Such misclassification can occur in otherwise asymptomatic never-smokers, starting at about age 45–50 (77). It is not fully understood how FeNO varies with age in healthy individuals. One study has demonstrated three distinct phases in the evolution of FeNO throughout the age range 6–80 years (78). FeNO values increased linearly between 6 and 14 years of age in girls and between 6 and 16 years of age in boys. After that, FeNO levels plateaued in both genders until age 45 years in females and age 59 years in males, when they started to increase linearly again. This increase continued until age 80.

### Smoking and Asthma Diagnosis

A smoking history should always be obtained as regardless of the underlying disease smoking cessation advice should be given. Current or past smoking, conventionally at least a 20 pack year history, opens up the potential diagnosis of a smoking asthmatic, COPD or ACO. To a certain extent these labels are arbritary and of more importance is the identification of underlying treatable traits (79). Active smoking will lower FeNO measurements (80) and any trial of treatment with either ICS or OCS may be affected by the impact of smoking on the mechanism of action of corticosteroids (81). Current smoking should not have any impact on blood eosinophils, spirometry, or bronchodilator reversibility.

### Occupational Asthma

Asthma either caused by occupation or aggravated by occupation should always be considered in people diagnosed with asthma during their working lives. Studies have suggested that up to 10% of people diagnosed with asthma in adult life have an occupational cause (82). Occupational asthma can be simply screened for in primary care by asking whether asthma symptoms improve at the weekend or when on holiday (83). If the answer to either of these questions is yes then a full occupational history including exposures should be obtained. The diagnosis of occupational asthma is extremely important given that moving away from the relevant exposure may allow for their asthma to be cured, providing that this occurs in a timely fashion. Everyone with occupational asthma should be referred to a specialist occupational lung disease unit who will perform detailed peak flow monitoring, potentially skin prick tests for the relevant allergens and on occasion challenge testing.

## Summary

There is definite consensus among adult physicians and pediatricians that asthma is a heterogeneous disease underpinned by numerous pathophysiological mechanisms. It is no longer acceptable to make a diagnosis in a patient of any age simply from a history and physical examination and by assessing a response to a trial of therapy. Young preschool aged children may not be able to undertake tests of lung function and inflammation that require voluntary maneuvers, but they can have assessments of atopy and blood eosinophils to help make a diagnosis and guide therapy. Children and adults above 5 years should all have at least some objective confirmation to support the diagnosis using lung function tests and non-invasive assessments of airway inflammation using exhaled nitric oxide. These tests can be undertaken in a primary or secondary care setting providing adequate education and training in the use and interpretation of the equipment is made available to health professionals. There is no gold standard test, but we now know that to avoid under diagnosis, over diagnosis and misdiagnosis, it is essential to undertake objective tests to support a diagnosis of asthma and to identify treatable traits of the airway disease.

## Author Contributions

All authors listed have made a substantial, direct and intellectual contribution to the work, and approved it for publication.

### Conflict of Interest Statement

The authors declare that the research was conducted in the absence of any commercial or financial relationships that could be construed as a potential conflict of interest.
